# The Physiology of the Human Brain

**Published:** 1850-07-01

**Authors:** William Senhouse Kirkes

**Affiliations:** Registrar, and Demonstrator of Morbid Anatomy at St. Bartholomew's Hospital


### THE PHYSIOLOGY OF THE HUMAN BRAIN.

By William Senhouse Kirkes, M.D., Registrar, and Demonstrator of Morbid Anatomy at St. Bartholomew's Hospital.

One of the most striking features by which the animal organizations are distinguished from vegetable, is the possession by the former of a set of functions superadded to, and exercising control over, the functions of mere organic life, such as digestion, respiration, secretion, and whereby, moreover, animal organisms are rendered conscious of the relations of external things, and become capable of a sentient enjoyment of existence. These additional functions are found to be exercised in all but the lowest order of animal life, by means of a peculiar kind of organic tissue which constitutes what is called a nervous apparatus, or system. In the very lowest forms of animated beings, such as many Infusoria, the more simple Entozoa, the Sponges, Polyps, Hydatids, and some others, no distinct nervous tissue can be discerned. Yet as these creatures manifest many of the qualities belonging to the nervous system in higher animals, such as consciousness, sensation, &c., the conclusion is formed that, in these lowly organized beings, the nervous function is in some manner diffused (as it were), through the entire body, and is possessed by the several tissues in virtue of a peculiar molecular condition. So soon, however, as a distinct nervous apparatus becomes apparent in the animal body, it is found to consist of fibres conveying impressions to and from nervous centres, or ganglia, whose office is to take cognizance of the impressions received, and to originate impulses for the performance of certain definite operations. And it is also found, as a general rule, that the rank which the several species occupy in the scale of animal creation, is mainly determined by the comparative size (in relation to the rest of the body), and by the complexity of the structure and arrangement of the chief nervous centres. In man, and all the vertebrate animals, the principal elements of the nervous apparatus are grouped together to form the great cerebro-spinal axis, which, though consisting of numerous segments, so intimately connected as to form but one organ, is yet, for purposes of description, commonly divided, as its name implies, into two parts; the one situated within the vertebral or spinal canal, and called the spinal cord; the other contained within the cavity of the cranium, and named the encephalon, or brain. It is with the functions of the latter that we have here to deal. In investigating the physiology of this organ, it will be necessary to consider it as consisting of four principal parts—namely, the cerebrum, cerebellum, pons varolii, and medulla oblongata; and each of these parts must be considered, both in the relation which it bears to the whole, and in relation to its individual office, as an independent nervous centre.

In considering, then, the physiology of the medulla oblongata, to which our attention shall be first directed, it will be desirable, before speaking of its individual functions as a distinct nervous centre, to say something of its anatomical relations.

Situated between and connecting together the brain and the spinal cord, the medulla oblongata is obviously the channel through which all communications between these two main portions of the cerebro-spinal axis are effected. Its structure is made up principally of bundles, or columns, of fibres, continuous with corresponding columns in the spinal cord, and endowed with the same general functions, the anterior columns transmitting motor, the posterior sensitive impressions. The similarity in function of the continuous parts of the medulla oblongata and spinal cord, has been proved by experiments. Division of one anterior pyramidal tract of the medulla oblongata was found, by Magendie, to be followed by complete loss of motor power over one half of the body, while its sensation seemed to be unimpaired. In Longet's experiments, irritation of the anterior pyramids was followed by no manifestation of pain, while the slightest touch of the restiform bodies elicited signs of most acute suffering. Moreover, a case is mentioned by Lebert, in which an affection of the anterior portion of the medulla oblongata was attended by great impairment of the power of motion, while sensation was unimpaired. There is no doubt, therefore, respecting the general identity in function, as well as in structure, between the medulla oblongata and the spinal cord; but, were further proof wanted, it might be found in the fact, that nerves of similar function arise from similar parts in these two portions of the cerebro-spinal mass. Thus the motor third and sixth pairs of cerebral nerves, which supply all the muscles of the eye but one, arise from the anterior pyramids of the medulla oblongata; the motor hypoglossal, which supplies the muscles of the tongue, arises from the groove between the anterior pyramids and the olivary tracts; this groove being continuous with that from which the motor roots of the spinal nerves emerge. The motor facial and the fourth nerves, the former supplying the muscles of the face, the latter the trochliaris muscle of the eye, arise from the lateral and round tracts, which are formed of fibres continuous with the anterior and lateral columns of the cord; while, from the front of the restiform tracts, which are continuous with the posterior or sensitive columns of the cord, arise the roots of the sensitive glosso-pharyngeal and pneumo-gastric nerves. But, although the fibres of the anterior and posterior columns of the spinal cord are, in one sense, continuous with those of the corresponding tracts of the medulla oblongata, yet the majority of the fibres of each anterior pyramid of the medulla oblongata, instead of passing into the anterior columns of the corresponding side of the cord, cross over to mingle with the anterior and lateral opposite half-fibres of the cord. Hence it is probable that the majority of motor impulses, originating in the brain, and transmitted through the medulla oblongata, pass down the opposite side of the spinal cord, and thus act upon the muscles of the opposite sides of the body. By this decussation of the motor fibres, the phenomenon of what is termed cross-paralysis—that is, loss of motion on the side opposite to that on which the effusion of blood in cerebral apoplexy has taken place, is naturally explained. There is, however, no appearance of the posterior, or sensitive fibres, decussating: so that an equally satisfactory explanation of the fact, that the paralysis of sensation, like that of motion, is also on the opposite side of the body, cannot yet be given.

Besides containing the parts which connect the brain and the spinal cord, and which serve to transmit impressions from one to the other, the medulla oblongata has, by virtue of the gray or vesicular matter in its composition, independent functions as a nervous centre, and these of very high importance, since upon them depend the performance of the processes of *respiration and deglutition,* so immediately necessary to the maintenance of life. Every part of the brain may be gradually sliced away, yet, provided the medulla oblongata be left uninjured, life will continue for a considerable time, and the respiratory movements go on uninterruptedly. So also may respiration and other signs of life continue when the spinal cord is successively cut away from below upwards, as high as the point of origin of the phrenic nerve; or, in birds and reptiles, which possess no diaphragm, even so high as the medulla oblongata, but the instant that organ is wounded, life ceases. In cold-blooded animals, both the brain and spinal cord have been successively removed, leaving only the medulla oblongata, and yet the animals have lived. When the medulla oblongata alone is wounded, the rest of the nervous system being left uninjured, life ceases at once, for the respiratory movements stop, and the animal dies asphyxiated. Experiments proving these things have been made again and again, especially by Legallois and Flourens: and additional proof has been repeatedly furnished by injuries and disease in the human subject. Thus Sir Charles Bell and others have recorded instances in which instantaneous death ensued on injury to the medulla oblongata: and it is to injury of the same part, or of that portion of the cord connecting it with the phrenic nerve, that the fatal termination of cases of fracture or displacement of the upper cervical vertebra is due.

This presiding power over the important act of respiration seems to reside, not in every portion of the medulla oblongata, but only in the interior of that part whence the pneumo-gastric nerves originate. For, it is said, that by careful management, the medulla oblongata may be divided to within a few lines of that point, and the exterior even removed, without the respiratory action ceasing; which, however, it does, the instant this vital part is wounded. It must not, however, be supposed, that because the pneumo-gastric nerves arise from this part, it is their injury that causes death in such cases, for both pneumo-gastric nerves may be divided, even close to their roots, without any other immediate evil consequence than retardation of the respiratory movements ensuing. The explanation seems to be, that from this point of the nervous centre issue those motor impulses which lead to the co-ordinate and adopted movements whereby the muscular act of respiration is carried on. Injury to this part at once puts an end to the production of fresh impulses, and respiratory movements consequently cease.

As well as being the centre of respiration, the medulla oblongata is also the centre whence proceed the motor impulses which enable the muscles of the palate, pharynx, and œsophagus to produce the successive co-ordinate and adapted movements necessary to the act of *deglutition.* Thus the power of swallowing continues when every other part of the brain except the medulla oblongata is destroyed, but ceases directly this latter is wounded, and it does so even though every other part of the nervous system is left intact. The act of deglutition is fully performed also in anencephalous monsters, and in marsupial embryos before the brain is developed.

Although the medulla oblongata presides over two of the most important functions in the body, viz. respiration and deglutition, and thus occupies the highest rank in relation to animal life; yet, viewed in relation to the mind, it has no claim to be considered as possessing any power of either perceiving impressions, or of giving issue to the dictates of the will, much less of being concerned in any of the higher intellectual arts. The movements which ensue after the whole of the brain, excepting the medulla oblongata, has been removed, are entirely of the reflex or involuntary kind, and appear totally independent of perception or volition. There are no manifestations of pain when the animal is injured, neither do voluntary muscular movements ensue. On proceeding, however, to the portion of brain which comes next in order to the medulla oblongata, viz.: the mesocephale, or *Pons Varolii,* we find the earliest traces of the faculties of sensation and of volition. Affording, in its anatomical structure, the several means—1st, by which the cerebrum is connected with all the tracts of the medulla oblongata, except the restiform and lateral; 2nd, by which the cerebellum is connected with these two tracts; and 3rd, by which the two hemispheres of the cerebellum are connected with each other; the pons, viewed in relation to its conducting power, obviously serves to transmit impressions between the several parts which, by its means, are brought into connexion with each other. The difficulty, or even impossibility, of determining with certainty anything respecting the various impressions conveyed along its several bundles of component fibres, renders it useless to state the results of experiments on the pons, so far as they relate to such points. But in regard to the functions of the pons as a *nervous centre,* the experiments of Flourens, Longet, and others, afford more satisfactory results, and render it probable that this part of the brain is capable of exercising some of the mental faculties.

Thus Longet, experimenting on rabbits and puppies, found, that when he had removed the whole of the cerebrum, including the corpora striata, optic thalami, and also the cerebellum, leaving the pons and medulla oblongata alone entire, the animal cried out when its tail was pinched, and attempted to remove with its paws irritating substances held to its nostrils. These manifestations of sensation and volition ceased directly the pons was destroyed. It is certainly possible, that the actions in question proceeded from a high order of reflex power, and therefore, do not conclusively establish that the pons is an organ through which the mind can receive and transmit impressions independently of the rest of the brain; but the low degree of the reflex power possessed by warm-blooded animals in general, and its subjection in them to the will, make it probable that the conclusion arrived at by MM. Flourens and Longet is correct.

The functions of the *cerebellum* may be considered in relation, 1st, to sensation; 2nd, to voluntary motion; and 3rd, to the instincts and higher intellectual acts. With regard to sensation, it appears strange, that although the highly sensitive restiform tracts of the medulla oblongata (irritation of which causes acute suffering) pass directly into the cerebellum, yet no pain ensues when the cerebellum itself is wounded,—indeed, the whole of it may be cut away without any signs of pain being elicited. So also when this portion of the brain is diseased in the human subject, there is usually no great amount of accompanying pain, and no loss or disorder of sensibility. The functions of the special senses also seem to be unimpaired by its removal; animals in which this experiment has been performed being able to see, hear, and smell, as well as perceive pain, apparently as perfectly as before. Hence it appears that the relation which the cerebellum bears to sensibility is at least of a very feeble kind.

In regard to motion, the cerebellum occupies a much higher position. Although belonging to the motor apparatus, yet the results of experiments and of disease show, that it cannot be regarded as a *source* of voluntary movements, but as a nervous centre, endowed with the faculty of controlling and regulating voluntary muscular actions, and of combining and co-ordinating the movements of the various muscles and groups of muscles, to the accomplishment of a definite and purposed end. The facts in support of this view of the co-ordinating faculty of the cerebellum are numerous; but it is mainly supported by the result of experiments performed by Flourens on birds and other animals, and since repeated and confirmed by Hertwig, Longet, and others. Removal of the superficial layers of the cerebellum in birds, was found to induce feebleness and want of harmony in the muscular movements; when the middle layers were reached, the movements became violent and more irregular; and by the time the whole of the organ had been cut away, the animals had completely lost the power of performing any combined and regular muscular action, such as flying, walking, or standing. They would perform irregular movements, and when placed on the back, were unable to change their position, though they endeavoured to do so. Perfect consciousness seemed to remain, for the animals in this state could see blows aimed at them, and endeavoured to avoid them. The results of these mutilations—which seem to prove that the chief office of the cerebellum is the co-ordination of voluntary movements—are in accordance with the information furnished by the comparative anatomy of the organ. For the tables of M. Serres show that, in the four great divisions of vertebrate animals, those species whose natural habits require varied and complex muscular movements have the cerebellum largely developed, in proportion to the spinal cord.

Although the foregoing is the generally adopted and most probable view of the office of the cerebellum, yet it should be mentioned, that other functions have been assigned to it by different physiologists. According to the theory of M. Foville, the cerebellum is the organ for the perception of muscular sensibility—that is, of the peculiar sensation by which the mind is informed of the actual condition of a muscle, especially as regards its state of contraction or relaxation. It is by this sensibility that we are not only made conscious of the morbid sensations of fatigue and cramp, but acquire a knowledge of the distance, weight, and resistance of bodies, as estimated by the muscular effort of which we are conscious in measuring, moving, or raising them. The hypothesis that the cerebellum is the organ through which the mind acquires this knowledge, certainly derives nearly as much support from the facts already mentioned, in speaking of the co-ordinating power of the cerebellum, as is afforded for the co-ordinating theory itself. Indeed, the two theories seem to admit of being reconciled with each other: for it is not improbable that the cerebellum may be the organ through which the mind is informed of the state and position of the muscles, and through which, at the same time, the will may be definitely and aptly directed to them, so as to produce combined and co-ordinate movements. It is well known that the followers of Gall and Spurzheim consider the cerebellum to be the seat of the generative instinct.

It were out of place here to enter at any length into this subject, but it may be desirable to mention the principal facts advanced in support of this theory, and some of the many objections which have been urged against it. In the first place, several cases are quoted in which loss of sexual power, and even atrophy of the testes, are asserted to have been the consequence of injury to the cerebellum: but this proves nothing to the point, unless it be shown that the loss of the sexual passion followed immediately after the injury to the cerebellum, and preceded the atrophy of the testes; for it is at least quite as probable that the loss of this power was owing to the wasting of the testes, and that this wasting might be caused by some connexion, in the process of nutrition, between the testes and the cerebellum, similar to that existing between the testes and the hair, larynx, and other parts. In the second place, cases have been adduced in which great excitation of the sexual organs has accompanied disease of the cerebellum: but similar phenomena have also been observed in injuries and diseases of the medulla oblongata, or of the upper part of the spinal cord. Again, it has been said that the powers of the sexual instinct in different persons may be estimated by the comparative development of the region of the cerebellum; or, in other words, that the size of the cerebellum determines the strength and amount of the sexual passion. Against this statement craniological facts might be adduced, but there are facts of another kind which carry more weight. Thus, absence of the cerebellum may occur without loss of the sexual passion. Also, there is no proportion between the size of the cerebellum and the amount of sexual power in different animals: some animals with small cerebella, as frogs and toads, having the sexual passion exceedingly strong, while others, such as the cod-fish, which are provided with large cerebella, have the instinct extremely feeble. To the same point, also, is the fact, that the cerebellum of the domestic cock is not larger than that of the hen, although his sexual passion is obviously much greater than hers. Again, destruction of the sexual power in early life, as by the process of gelding, is not followed by diminution in the size and weight of the cerebellum; indeed, MM. Lassaigne and Leuret found that both the absolute and relative weight of the cerebellum in geldings even exceeded that of mares and stallions.

In considering the physiology of the *cerebrum*, the same order as that hitherto pursued may be continued, namely:—proceeding from the lower and simpler, to the higher and more complex parts. The cerebrum is connected with the mesocephale and medulla oblongata by means of the two *crura cerebri*, which are therefore the channels along which all nervous impressions passing through these nervous centres are transmitted. Division of both crura consequently cuts off all communication between the cerebrum and the parts below it: division of one crus causes a peculiar disorder in the movements of the body (owing to the loss of balance between the muscles of the two sides);—the animal turning round and round from the injured towards the sound side, as if from partial paralysis of the side opposite to the injury.

Situated on the upper part of the crura cerebri are the three pairs of small ganglia, namely: the *corpora geniculata externa* and *interna*, and the *corpora quadrigemina*. The functions of these several bodies may be considered identical; the office of the corpora geniculata being as yet undistinguished from that of the corpora quadrigemina, which are justly regarded as the special nervous centres for the sense of sight, being, indeed, the homologues of the optic lobes in birds, amphibia, and fishes. Removal of one of the corpora quadrigemina causes blindness of the opposite eye, removal of both wholly destroys the sense of vision. Blindness is also a common accompaniment of disease implicating these ganglia; and atrophy of them usually follows total loss of sight. Contraction of the iris ensues when the corpora quadrigemina are irritated; but when they are destroyed the iris remains permanently dilated. These facts prove the important relation of the corpora quadrigemina to the sense of vision. And this relation does not consist in the mere anatomical connexion of these ganglia with the optic tracts or roots of the optic nerves, but in their being the special organs in which the mind perceives all visual sensations. For it was shown by Longet's experiments, that when the cerebral hemispheres of a pigeon are removed, and its optic thalami and optic lobes, or corpora quadrigemina, are left, the power of vision is scarcely, if at all, impaired; for the iris contracts, on a light being held to the eye, and when the light is withdrawn the bird moves its head as though it were watching it. No other sense than that of sight seems to suffer by the removal of the corpora quadrigemina: the general movements are somewhat disordered, but no more than might result from giddiness and partial loss of sight. With regard to the intellectual faculties and the affections, there is no reason for supposing that the corpora quadrigemina hold any special relation to any of these. It is not improbable, however, that these bodies have some other, perhaps secondary, function, beside that relating to vision; for, as a general rule, neither their absolute nor their relative size in different animals, bears any direct proportion to the acuteness or extent of their several powers of vision.

The *optic thalami* have not, as their name would imply, a very important relation to the sense of vision, though they probably have some share therein, since part of the fibres of the optic tract may be traced to their surface. A slight impairment of vision was lately noticed by the writer in a case in which an apoplectic clot was found situated near the surface of one of the optic thalami. Diseases and experiments, however, prove but little on this point: what they both show is, that injury of these nervous masses is productive of considerable disturbance of the movements of the body. Destruction of one thalamus causes results similar to those induced by division of one crus cerebri, as before mentioned.

According to both Schiff and Longet, the two cerebral hemispheres and corpora striata can be removed, and the animal still possess the power of standing and walking; but the instant one of the optic thalami is destroyed, the animal either falls down paralyzed on the opposite side, or commences the rotatory movement. A partial or complete loss of power on the opposite side of the body is also the most frequent effect of apoplexy affecting one of the optic thalami.

Next in order to the optic thalami come the *corpora striata*:—Respecting the function of these bodies, however, nothing certain can be said. Experiments and disease prove only that they have no particular relation to voluntary motion or general sensation, or any special sense. The experiments of Magendie, Schiff, and others, showed, that when they were removed in rabbits, sensation seemed to be unimpaired, and the power of movement entire. The results, therefore, which these and other experiments on the same structure furnish, are simply of a negative kind. It is almost unnecessary to remind the reader of Dr. Todd's hypothesis—"That the centre of *volition* consists primarily of the corpora striata."

The only portions of the brain which remain to be considered, are the cerebral hemispheres. The several parts of which we have already treated, together with the spinal cord and sympathetic system, are endowed with all the functions requisite for the direction and government of the various processes by which animal life is maintained; also for the perception of the several sensations, and for the direction of such instinctive and habitual movements as do not require the exercise of attention, judgment, memory, or any other intellectual act. The medulla oblongata and spinal cord can produce none but involuntary and unintentional movements; but as we rise to the parts above the medulla oblongata, *e. g.*, the mesocephale we find organs undergoing such conditions as the mind can perceive, and capable of being excited to voluntary and orderly operations. Although these higher organs are endowed with sensation, and are capable of receiving the direct mandates of the will, it is, however, only in relation to the lower and more instinctive actions, that the will is thus manifested upon them. The mandates of the will for all the more complex actions issue from the cerebral hemispheres, which are also the parts endowed with the higher faculties of the mind. Occupied with these exalted functions, the cerebral hemispheres probably take but little part in the functions over which the portions of the brain already treated of, preside. These portions, which commonly pass by the name of the cerebral or sensory ganglia, seem to possess the power of discharging their functions independently of any influence derived from the cerebral hemispheres: for, as we have seen in several instances, the removal of the hemispheres has not been followed by more than a temporary arrest of the functions discharged by these sensory ganglia. That the ganglia do naturally discharge their functions independently of the cerebral hemispheres, is made probable also from the fact that all the cerebral nerves are in direct communication with them, and are connected with the cerebral hemispheres only through the medium of the highest of them. Moreover, the homologues of these several ganglia, *i. e.*, the corpora quadrigemina, the optic thalami, corpora striata, and the olfactory lobes or ganglia, maintain, in the descending scale of vertebrate animals, a large size, and are proportionate to the perfection of the special senses; while the cerebral hemispheres, on the other hand, gradually diminish in their proportional development till, in the highest fish, they are not larger than the largest of these ganglia, and in the lower fish are not larger than the optic, or olfactory lobes. In the same descending series the higher intellectual faculties seem to diminish commensurately with the decrease of the cerebral hemispheres; but there is no corresponding decrease of those lower powers of the mind, of which these ganglia at the base of the brain are supposed to be the organs. Neither, perhaps, can any such diminution be traced in those conditional acts or expressions, which belong to the common instincts that all animals manifest in different degrees,—such as fear, anger, jealousy, &c.; of which, also, it is not improbable the cerebral ganglia may be the organs: although this can only be surmised so long as we know so little of the emotions to which lower animals are subject.

It appears, then, that all the necessary actions of the animal body are determined and regulated by parts of the nervous system distinct from the cerebral hemispheres, and that, therefore, no other functions remain to be discharged by these larger portions of the brain in man, and the higher mammalia, than those connected with the intelligence and will. They may, indeed, be considered as the material organs by which the mind performs all sensuous operations—the mind, in things purely spiritual, and beyond the cognizance of the senses, (such as are included under *conscience* and *pure reason,*) being probably exercised independently of the brain. The cerebral hemispheres, considered conjointly and as a single organ, may be said to be the instrument by which the intelligence is brought into relation with external objects, and by which the mind—

1st. Possesses an intelligent *consciousness.*

2nd. Has the voluntary faculty of exercising its powers in a given direction (*attention, volition*).

3rd. Perceives all physical sensations, and forms ideas (*perception, conception*).

4th. Retains ideas; and reproduces them, either simply or in combination (*memory, imagination*).

5th. Classifies and compares ideas (*association, judgment*).

The foregoing are the fundamental principles, or faculties, of the human intellect, and seem to be exercised in common through the medium of the cerebral hemispheres. That the cerebral hemispheres are the organs through which the mind is enabled to manifest itself in relation to external things, is made probable by several circumstances, in addition to those already mentioned. Thus it is found that any severe injury of the hemispheres usually deprives a person at once of all power of manifesting any mental faculty. Also, that feebleness of the intellect is a general attendant upon congenital deficiency, and also upon certain morbid changes in the hemispheres. Again, as a general rule, the higher the mental faculties are developed in the vertebrate animals, and in man, at different ages, the larger do the cerebral hemispheres become, in comparison with the rest of the cerebro-spinal system: the development of no other part of the nervous system bearing any corresponding proportion to the increase of the mental faculties.

The expression that the cerebral hemispheres furnish the medium through which the mind manifests itself externally, naturally implies that the mind must be regarded as an immaterial principle, not dependent on the brain for its existence, but merely using the brain (as an artist uses the implements of his art), for its instrument, whereby it gains knowledge of external things, and manifests its own relation to them. Such a principle would itself remain unchanged in the case of injury or disease: its external manifestations at such times being interrupted by the imperfection of the instrument through which these manifestations are effected, but resumed when its instrument, the brain, should be sufficiently restored to allow of its being again employed.

The mode in which the mental principle operates in its connexion with the brain, is still quite obscure. It appears certain, however, that, for all but the highest intellectual acts, one cerebral hemisphere is sufficient: for cases have not unfrequently occurred, in which one hemisphere was so altered in structure as to be apparently incapable of discharging its functions, yet in which no mental defect was observed. Here the remaining healthy hemisphere probably discharged the functions of both, and sufficed for the performance of all the ordinary mental acts, though it would probably have been found insufficient for the higher intellectual faculties, had its power in relation to these been tested. In health, the mind combines the impressions received by the two hemispheres, and produces from them single ideas, like as in healthy vision the impressions on the two retinæ give rise to a single perception. In certain forms of disease, however, in which, perhaps, one or both hemispheres are disordered, the same object may produce two separate sensations, and suggest simultaneously different ideas; or, at the same time, two trains of thought may be carried on, by the one mind acting, or being acted upon differently in the two hemispheres. Much of the incoherence of delirium and dreaming seems to be the result of this want of harmony in the action of the two hemispheres of the brain; and it is principally from phenomena of this kind that the theory of the duality of the mind has arisen.

The sensational impressions and voluntary impulses which pass to and from the cerebral hemispheres, are carried across the middle line, owing to the decussation of fibres already alluded to; hence the loss of sensation, or of voluntary movement, resulting from injury to either hemisphere, are observed on the side of the body opposite to that on which the injury is inflicted.

So far the cerebral hemispheres have been regarded as a single organ, of which all parts are equally appropriate for the exercise of each of the mental faculties. There are, however, several facts which make it probable that each special faculty of the mind has a special portion of the brain appropriate to it as its own proper organ. This view is in accordance with what is observed in the physiology of other compound organs or systems in the body, in which each part has its special function—as in the digestive system for example, where the stomach, liver, pancreas, and each of the other organs, performs its own particular part in relation to the general process of digestion. This view is supported also by the facts that each of the several faculties of the mind admits of being manifested independently of the rest; is capable of being educated beyond the others by exercise; seems to exist naturally in different persons in degrees which bear no definite proportion to the other faculties; may be quiescent in sleep, while one or more of the other powers may be active; and seems to admit of being disordered exclusively, or in greater degree than the rest, in some of the various forms of insanity. These, which are some of the more striking arguments brought forward by the founders of the theory on which the system of phrenology is built, certainly seem to indicate that the manifestations of the several faculties of the mind are effected through the instrumentality of as many separate and special organs in the brain. Yet, although this theory itself may be safely admitted, it by no means follows that the extensive system which has been erected upon it is to be credited to a like extent; indeed, the objection to the soundness of a system which professes to have determined all the primitive faculties of the mind, as well as the special locality in the brain assigned to each, are too numerous and obvious to warrant its acceptance.

In this short review of the physiology of the brain, several of the minor portions of the cerebral mass, to which names are given, have been omitted, principally because so little could be said respecting their functions. Among these omissions are the several commissures—*e.g.*, the fornix, and corpus callosum, the functions of which probably consist simply in connecting the actions of the parts between which they are severally placed. The corpus callosum, situated between and connecting together the two cerebral hemispheres, and thus probably enabling the two sides of the brain to act in concert, seems, however, to have this function exercised only in the higher acts of the mind; for several cases have been recorded in which it was absent, or very defective, yet in which no evident mental deficiency was observed.

The functions of the pineal and pituitary bodies are at present quite unknown. It is even doubted whether they have any special relation to the nervous system, and whether they ought not rather to be classed among the glands without ducts, as the spleen and supra-renal capsules, with which in structure they are said to be closely identical.

